# Anesthesia and perioperative care management in patients with Dengue Fever: considerations and challenges

**DOI:** 10.1016/j.bjane.2024.844511

**Published:** 2024-05-08

**Authors:** Lorena Ibiapina Mendes de Carvalho, Liana Maria Tôrres de Araújo Azi, Plinio da Cunha Leal, Michelle Nacur Lorentz, Luis Antonio dos Santos Diego, André P. Schmidt

**Affiliations:** a Hospital Prontomed, Departamento de Anestesiologia, Teresina, PI, Brazil; b Hospital Unimed Primavera, Teresina, PI, Brazil; c Hospital Universitário Professor Edgard Santos, Departamento de Anestesiologia, Salvador, BA, Brazil; d Universidade Federal da Bahia (UFBA), Salvador, BA, Brazil; e Hospital São Domingos, Departamento de Anestesiologia, São Luís, MA, Brazil; f Universidade Federal do Maranhão (UFMA), São Luís, MA, Brazil; g Hospital Biocor / Rede D'or, Nova Lima, MG, Brazil; h Universidade Federal Fluminense (UFF), Departamento de Anestesiologia, Rio de Janeiro, RJ, Brazil; i Hospital de Clínicas de Porto Alegre (HCPA), Serviço de Anestesia e Medicina Perioperatória, Porto Alegre, RS, Brazil; j Universidade Federal de Ciências da Saúde de Porto Alegre (UFCSPA), Santa Casa de Porto Alegre, Serviço de Anestesia, Porto Alegre, RS, Brazil; k Hospital Nossa Senhora da Conceição, Serviço de Anestesia, Porto Alegre, RS, Brazil; l Universidade Federal do Rio Grande do Sul (UFRGS), Programa de Pós-graduação em Ciências Pneumológicas, Porto Alegre, RS, Brazil; m Universidade Federal do Rio Grande do Sul (UFRGS), Programa de Pós-graduação em Ciências Cirúrgicas, Porto Alegre, RS, Brazil; n Faculdade de Medicina da Universidade de São Paulo (FMUSP), Programa de Pós-Graduação em Anestesiologia, Ciências Cirúrgicas e Medicina Perioperatória, São Paulo, SP, Brazil

Dengue is an arbovirus infection transmitted through *Aedes aegypti* mosquitoes that annually impacts millions worldwide. Its causative agent is the Dengue virus (DENV), with four serotypes in humans (DENV 1-4).^1^, ^2^, ^3^, ^4^, ^5^, ^6^ The pathogenesis of Dengue initiates upon viral entry into the bloodstream, targeting and infecting key cells such as endothelial cells, macrophages, and monocytes. This provokes an immune response and release of a variety of inflammatory mediators. While aimed at controlling viral replication, the response significantly influences the clinical spectrum of the disease, from mild to severe presentations.^1^

Dengue exhibits widespread distribution in over 100 countries, mostly in tropical and subtropical regions, including Brazil. Approximately half of the world's population is at risk of Dengue infection, underscoring its status as a critical public health issue.^2^^,^^6^

This editorial aims to update clinical manifestations, diagnosis, and classification of Dengue Fever, and clarify anesthesia and critical care management in the perioperative period. Briefly, the search strategy was published literature until April 13, 2024 in PubMed, Scopus, Lilacs and Google Scholar databases, and keywords were: (("dengue") OR ("dengue infections")) AND (("anesthesia") OR ("surgery") OR ("surgical complication") OR ("management") OR ("perioperative period") OR ("postoperative") OR ("pregnancy") OR ("intensive care")). We included articles in English, Portuguese, and Spanish and evaluated the full text of the most relevant records. The authors’ opinions were considered for doubtful topics and in the lack of specific literature, but do not represent formal guideline recommendations.

## Clinical manifestations

**Figure 1:**
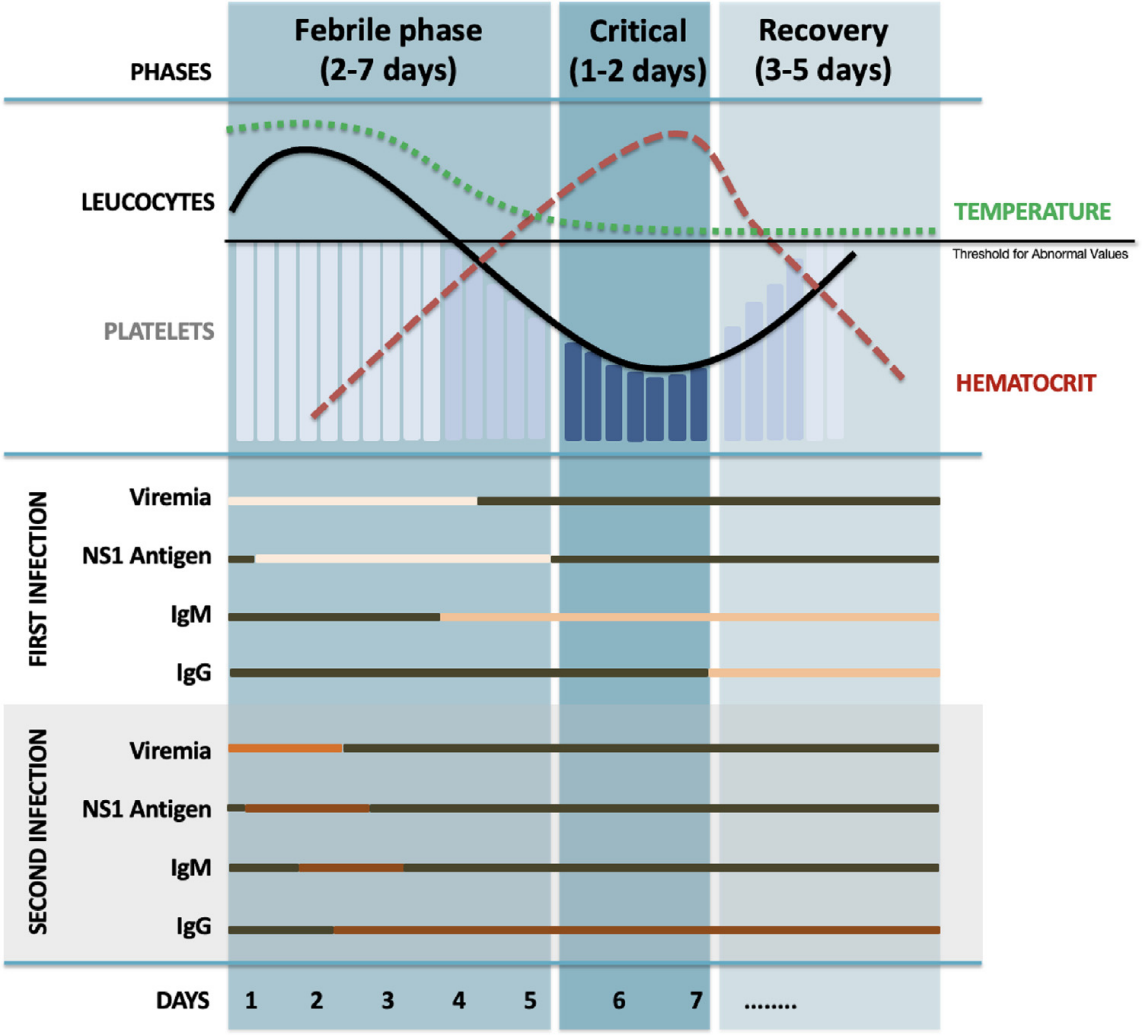
**Correlation between clinical phases of Dengue infection and associated clinical and laboratory findings.** Adapted from: Tejo AM, Hamasaki DT, Menezes LM, Ho YL. Severe dengue in the intensive care unit. J Intensive Med. 2024;4(1):16-33.

Dengue virus infection can manifest as asymptomatic or as a systemic and dynamic disease with a wide clinical spectrum, which can progress to death. Clinically, it unfolds into three phases: febrile, critical, and recovery (Fig. 1).

The febrile phase is characterized by sudden high fever (39–40°C), severe headache, asthenia, myalgias, arthralgias, retro-orbital pain, and rash (occurring in 50% of the cases, predominantly maculopapular, affecting face, trunk, and limbs). Typically, it lasts 2–7 days. This phase may be mistaken for other febrile illnesses, and in most patients, there is a gradual improvement, with a return to general well-being.^2^^,^^7^

The critical phase begins after fever resolution and is marked by plasma leakage, fluid accumulation, respiratory distress, and severe bleeding. Close monitoring for warning signs is required in this phase, indicating potential progression to Severe Dengue and warranting immediate medical intervention. Warning signs include severe abdominal pain, persistent vomiting, rapid breathing, bleeding gums, fatigue, and blood in vomit or stools. Additional severe manifestations include hemorrhage and dysfunction of critical organs, including heart, lungs, kidneys, liver, and central nervous system.^2^^,^^6^ Patients who have gone through the critical phase experience gradual reabsorption of the extravasated content, leading to progressive clinical improvement over the following 48 to 72 hours (recovery phase).^2^^,^^3^^,^^6^

The most recent Dengue severity classification (WHO, 2009) divides cases into 3 groups: A) Dengue Fever without warning signs; B) Dengue Fever with warning signs; C) Severe Dengue.^6^ Severe Dengue is defined by one or more of the findings: 1) shock or respiratory distress, 2) bleeding, and 3) severe organ damage.^2^^,^^3^^,^^5^^,^^6^ Patients with secondary infections are at risk of severity. Several co-existing conditions increase the risk of Severe Dengue: pregnancy, diabetes, hypertension, extremes of age (< 1 year and > 60 years), hemolytic anemia, chronic renal or hepatic failure, asthma, chronic obstructive pulmonary disease, and use of anticoagulants.^2^^,^^6^^,^^8^

Differential diagnoses for Dengue include other infectious diseases, such as Malaria, Leptospirosis, Typhoid Fever, and Zika and Chikungunya viral infections, due to overlapping symptomatology. Dengue, Zika, and Chikungunya share the same vector and some clinical features. Serological assays and polymerase chain reaction (PCR) testing for Dengue virus infection confirm the diagnosis.^1^^,^^9^^,^^10^

**Figure 2:**
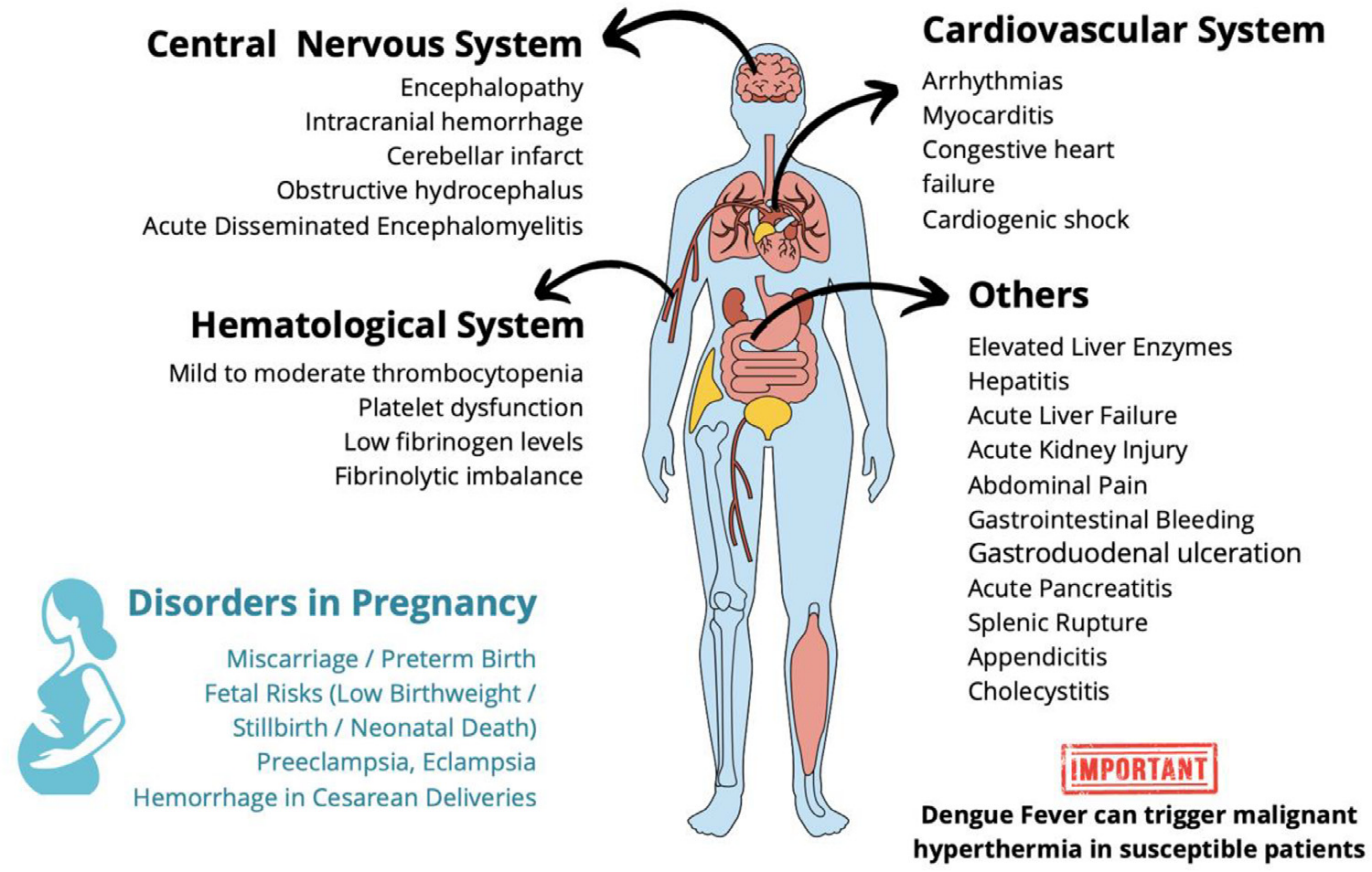
**Clinical manifestations of Dengue Fever across various systems** (original).

Dengue Fever can generate major organ dysfunction, mainly affecting coagulation, cardiovascular, pulmonary, central nervous system, and others (Fig. 2).

### Hematological system and coagulopathy

Coagulopathy, vasculopathy, platelet dysfunction (qualitative and quantitative), and imbalance between clotting and fibrinolytic systems are present.^11^ Low fibrinogen levels and prolonged activated partial thromboplastin time (aPTT) are the culprits for coagulopathy. Mild to moderate thrombocytopenia (considered a severity marker) occurs 3 to 7 days after infection and returns to normal levels on day 8 or 9 of infections in adults without Severe Dengue.^12^^,^^13^

### Cardiovascular

Cardiac manifestations of Dengue Fever can present with a mildly raised cardiac enzyme to severe myocarditis leading to congestive heart failure, arrhythmias, cardiogenic shock, and death.^14^ Myocarditis occurs in approximately 21% of Dengue Fever cases.^15^

### Central nervous system

Neurological involvement includes encephalopathy, intracranial hemorrhage,^16^ cerebellar infarction, and obstructive hydrocephalus.^17^^,^^18^ Encephalopathy affects from 0.5 to 6.2% of patients. Coagulopathy may cause cerebellar infarct, obstructive hydrocephalus, and spontaneous intracranial hemorrhage with spinal cord hematoma. Dengue patients have a slightly increased risk of non-vascular dementia.^19^

### Others

Hepatitis and elevated liver enzymes are frequent. Acute liver failure, encephalitis, myocarditis, and acute kidney injury (AKI) are less frequent but can occur.^15^ Among hospitalized patients with Severe Dengue, 3.3 to 4.8% develop AKI, with 14.1% requiring dialysis.^20^^,^^21^ Dengue Fever can also trigger malignant hyperthermia in susceptible patients.^22^

## Dengue fever and pregnancy

The Dengue virus is related to major consequences on pregnancy, ranging from abortion in the first trimester to severe maternal illness in the second and third trimesters. Cesarean deliveries are more frequent and adverse outcomes include hemorrhage, preeclampsia, and eclampsia.^12^ Dengue is also hazardous to the fetus (increased risk of miscarriage, stillbirth, and neonatal death), but no fetal malformations have been identified.^23^ Maternal-fetal transmission has been described. Preterm birth (< 37 weeks) and low birth weight were the most common adverse pregnancy outcomes.^24^

## Perioperative implications of Dengue Fever and anesthetic management

Severe Dengue causes fluid leakage, microvascular changes, and coagulopathy.^13^ Moreover, the immunological imbalance of Dengue Fever superimposes surgical trauma, leading to surgical complications and major postoperative adverse events, including hemorrhage, systemic inflammation, and shock.^13^^,^^21^

Generalized abdominal pain can be a true or apparent cause of acute abdomen in Dengue.^25^ Gastrointestinal bleeding, acute pancreatitis, and splenic rupture have been described.^25^ Acute appendicitis and cholecystitis can occur, due to direct viral invasion or extravascular leakage and serous edema.^25^^,^^26^ Neurosurgical emergencies are further challenges.^16^^,^^17^

## Preoperative evaluation

Preoperative evaluation of the Dengue Fever patient involves a detailed history comprising: 1) days of symptoms; 2) clinical phase; 3) warning signs; 4) severity and organic dysfunction, focusing on hemodynamic instability, plasma leakage, and bleeding potential; and 5) urgency of the case.^27^ In surgical patients, suspected Dengue cases must be confirmed by direct or indirect laboratory methods (Fig. 1), depending on clinical manifestations and local resources.

For hypertensive patients, discontinuation of diuretics and other antihypertensives in case of plasma leakage and shock is recommended. Vigilance of glycemic levels and discontinuation of oral hypoglycemic agents are warranted in diabetic patients.^5^ Statins should be interrupted during Dengue infection due to their potential to increase transaminases and creatine kinase levels.^5^

### Preoperative exams

Main preoperative exams in Dengue Fever and their respective findings include full blood count (baseline hematocrit and platelet counts), serum glucose and electrolytes, renal, hepatic and coagulation tests, thorax radiograph, point-of-care ultrasound (pleural effusion, B-lines in pulmonary edema, ascites, pericardial effusion), electrocardiogram (bradycardia, atrioventricular block, T-wave and ST-segment abnormalities), echocardiogram (ventricular dysfunction and myocarditis).^5^^,^^25^^,^^27^ Outcome and mortality markers are useful in high-risk and critical patients (admission APACHE II and SOFA scores, lactate, serum albumin, and procalcitonin levels).^7^

### Perioperative vaccination

Currently, there are two licensed Dengue vaccines: Dengvaxia (Dengvaxia, Sanofi Pasteur Inc., France) and Qdenga (TAK-003, Takeda, Japan).^1^ Both are tetravalent live attenuated vaccines targeting all four Dengue serotypes and approved for 9 years and older individuals from endemic areas, with previously confirmed Dengue infection.^1^^,^^9^^,^^28^ The Brazilian Ministry of Health has incorporated Qdenga as a public health policy since December 2023.^29^ Adverse effects in vaccinated patients during the perioperative period remain controversial, but there is an isolated recommendation to postpone elective procedures for 3 weeks after attenuated virus immunization and wait 7 days following surgery for vaccination.^30^^,^^31^ Yet, this is not a formal guideline, and surgery is not a barrier to immunization programs.

## Criteria to undergo elective surgery

Postponing elective surgery after Dengue Fever infection is a relevant but neglected topic. Limited evidence from case reports suggests significant postoperative complications due to Dengue, mainly hemorrhage and respiratory distress.^11^^,^^13^^,^^21^^,^^32^, ^33^, ^34^, ^35^ Hence, it is strictly advised to avoid surgery during acute Dengue infection, even in mild cases. This recommendation is supported by the existing literature and experience with perioperative Dengue Fever patients. Some experts recommend postponing elective surgery based on the anticipated progression and severity of the disease. Following a confirmed case of Dengue, with day 1 being the onset of symptoms, the suggested waiting periods are as follows: 14 days for group A patients (mild disease), 21 days for group B (patients with warning signs or co-existing conditions), and no specific recommendation for group C (Severe Dengue). However, clinical judgment is paramount, particularly when considering major or time-sensitive surgeries and the vulnerability of the patient.

Additionally, all health-care discharge criteria must be fulfilled: 1) clinical – no fever for the past 48 hours without antipyretics use and improvement in clinical status (general well-being, appetite, normal hemodynamic status, urine output and respiratory function, and absence of bleeding); and 2) laboratory - increasing trend in platelet count and stable hematocrit without the need for intravenous fluids.^6^

## Patients on anticoagulant or antiplatelet medication

The main concerns in managing antiplatelet and anticoagulant therapy in Dengue Fever are: 1) risk of thrombotic events, particularly in patients with coronary stents under dual antiplatelet therapy (DAPT); 2) acute bleeding and shock; and 3) low platelet count or rapidly declining trend.^36^

In cases of bleeding, it is imperative to discontinue antiplatelet and/or anticoagulant therapy promptly. Platelet transfusion or fresh frozen plasma may be necessary depending on the severity of bleeding. Hospitalization is recommended for close monitoring of thrombocytopenia in patients at high risk of thrombosis or with platelet counts < 50 × 10^3^ cells.μL^−1^.^3^

The Brazilian Ministry of Health offers specific recommendations for managing antiplatelet and anticoagulant therapy in Dengue Fever:^3^

**DAPT:** 1) It is generally advised to maintain DAPT in patients with conventional coronary stents implanted within the past month or drug-eluting stents implanted within the past 6 months. 2) If platelets < 30 × 10^3^ cells.μL^−1^, withholding DAPT is recommended.^3^ Some experts suggest considering discontinuation of DAPT at higher platelet counts (50 × 10^3^ cells.μL^−1^).^36^
**Anticoagulants:** 1) warfarin: if platelet count below 30 × 10^3^ cells.μL^−1^ - withhold; if between 30 and 50 × 10^3^ cells.μL^−1^ - initiate bridging therapy with unfractionated heparin (UFH); and if > 50 × 10^3^ cells.μL^−1^ – maintain warfarin therapy with regular monitoring of activated prothrombin time.^3^
**Direct thrombin inhibitors (DTIs) and direct factor Xa inhibitors (xabans**): if platelets < 50 × 10^3^ cells.μL^−1^, discontinue therapy, followed by hospitalization for bridging therapy with UFH. Bridging therapy should commence after 24 h from the last dose of dabigatran, rivaroxaban, apixaban, or edoxaban, or two times the half-life of the medication.

## Intraoperative anesthetic management

### Airway management

Plasma leakage in Dengue Fever can lead to airway edema and an increased risk of bleeding during manipulation and intubation, requiring careful attention to airway management.^27^ Additionally, patients may experience compromised pulmonary function, hypoxia, and respiratory distress. The risk of aspiration during induction is heightened in cases of persistent vomiting.^6^

### Anesthetic agents

In Severe Dengue, plasma leakage and increased capillary permeability are the key mechanisms of hypovolemic shock.^5^ Pharmacological repercussions of shock are: 1) reduced central compartment volume and clearance;^37^ 2) increased concentration and effect of intravenous anesthetics (propofol with the highest potency in shock, and etomidate the most stable);^37^ and 3) decreased minimum alveolar concentration of inhaled anesthetics.^38^ In Severe Dengue, rational anesthetics use is recommended; if possible, guided by processed electroencephalogram monitors, such as bispectral index. Baseline neurological assessment is paramount.

Hypoalbuminemia in Dengue Fever leads to an increased unbound fraction and higher plasmatic concentration of local anesthetics, resulting in a lower threshold for local anesthetic systemic toxicity (LAST), particularly in extreme age, comorbidities, and critically ill patients.^39^ Cardiovascular and neurological manifestations of Dengue Fever may complicate the diagnosis of LAST. Therefore, the use of local anesthetics requires caution, adhering to toxicity dose limits, and avoiding continuous blocks.

### Neuraxial anesthesia and invasive procedures

Neuraxial anesthesia is contraindicated in cases of active bleeding due to the risk of spinal or epidural hematoma. Special precautions are also necessary for invasive procedures: nasogastric tube insertion, bladder catheterization, airway manipulation, and the placement of central venous and arterial lines, which should be guided by ultrasound.^6^

A platelet count < 50 × 10^3^ cells.μL^−1^ is a formal contraindication for neuraxial anesthesia, gastrointestinal endoscopy, and major surgery (exceptions: neurological and ocular surgeries [100 × 10^3^ cells.μL^−1^], and central line placement [20 × 10^3^ cells.μL^−1^]).^40^ For epidural catheter placement or removal, a minimum of 75 × 10^3^ cells.μL^−1^ platelets is recommended.^40^ The risk of spinal epidural hematoma associated with a platelet count ≥ 70 × 10^3^ cells.μL^−1^ is likely to be very low in obstetric patients with thrombocytopenia.^41^ This reference may be applied to obstetric patients with Dengue Fever, but decisions regarding most cases are made on an individual basis.^42^^,^^43^ Moreover, functional platelet impairment is an additional hazard.^12^

## Hemodynamic and fluid management

The cornerstone to reduce morbidity and mortality lies in the early recognition of plasma leakage and supportive management.^1^^,^^2^ Severe Dengue patients must be admitted to the intensive care unit (ICU) and receive vigorous fluid expansion. Current evidence shows no significant difference between crystalloids and colloids for Dengue Fever; therefore, initial fluid expansion with crystalloids is recommended. Colloids, primarily 5% albumin, may be considered for cases of persistent shock, for instance, after three crystalloid boluses of 10-20 mL.kg^−1^.h^−1^ over 30 min to 1 h each.^5^^,^^7^^,^^9^^,^^29^^,^^44^ Clinical parameters indicating response to expansion include decreased tachycardia, improvement of blood pressure or pulse volume, return of capillary refill to < 3 s, level of consciousness, urine output ≥ 0.5 mL.kg^−1^.h^−1^, resolution of acidosis, and most importantly, decreased hematocrit.^2^^,^^5^ It is important to constantly reassess fluid response to mitigate fluid overload, especially in the early recovery period.^6^

## Patient blood management (PBM)

PBM is a well-established practice grounded in three major goals: optimizing erythropoiesis, minimizing blood loss, and managing anemia.^45^ PBM has been previously applied in the context of Zika virus infection^45^ and can be similarly employed for Dengue Fever to optimize patient care and reduce unnecessary transfusions.

In a randomized controlled trial in adult patients with Dengue Fever and ≤ 20 × 10^3^ platelets.μL^−1^, prophylactic platelet transfusion was not superior to supportive care in preventing bleeding and was associated with more adverse events (circulatory overload and allergic reactions).^46^

Bleeding in Dengue Fever is multifactorial and cannot be solely attributed to thrombocytopenia.^36^ It affects 20–60% of hospitalized Dengue patients^36^ and it is an indicator of disease severity. Hematocrit levels and curves are inverse to platelet counts in the critical phase (Fig. 1), and offer a practical guide for the optimal moment for procedures.^5^ Thus, the selection of blood components should directly address the underlying cause of coagulopathy: FFP (10 mL.kg^−1^), cryoprecipitate (1 unit per 10 kg), and vitamin K.^5^ Individualized transfusion strategies should be employed, guided by specific goals and using tools such as thromboelastography (TEG) and rotational thromboelastometry (ROTEM) when available.^45^

## Analgesia and adjuvants

Analgesia plays a crucial role in perioperative care, and a multimodal approach is currently the standard practice. However, it is important to tailor analgesic strategies to accommodate the specific limitations posed by Dengue Fever during invasive procedures. Even minor punctures can lead to significant hematomas in Dengue patients, making peripheral nerve blocks and neuraxial blocks challenging.^32^^,^^40^ Nonsteroidal anti-inflammatory drugs (NSAIDS) and acetylsalicylic acids should be avoided due to the risk of gastrointestinal bleeding and Reye's Syndrome.^2^^,^^6^ Similarly, intramuscular injections are discouraged in Dengue patients.^6^ Furthermore, corticosteroids lack evidence for efficacy in treating Dengue Fever.^5^

## Conclusions

The management of Dengue patients involves timely acknowledgment of risk predictors, warning signs, and laboratory markers to guide rapid interventions. Despite being a worldwide epidemic disease, Dengue Fever remains neglected in many aspects, including anesthetic and perioperative care management. Future research and evidenced-based guidelines are of foremost importance to better understand the implications of Dengue in anesthetic practice and improve outcomes.

## Conflicts of interest

The authors declare no conflicts of interest.
